# K-134, a Phosphodiesterase 3 Inhibitor, Prevents Brain Damage by Inhibiting Thrombus Formation in a Rat Cerebral Infarction Model

**DOI:** 10.1371/journal.pone.0046432

**Published:** 2012-10-23

**Authors:** Hideo Yoshida, Yuka Ashikawa, Shinsuke Itoh, Takashi Nakagawa, Akimune Asanuma, Sohei Tanabe, Yoshihiro Inoue, Hiroyoshi Hidaka

**Affiliations:** 1 Tokyo New Drug Research Laboratories, Kowa Company, Ltd., Tokyo, Japan; 2 D. Western Therapeutics Institutes, Inc., Nagoya, Japan; University of Münster, Germany

## Abstract

**Background:**

K-134 is a more potent antiplatelet drug with a selective inhibitory effect on phosphodiesterase 3 (PDE3) compared with its analogue, cilostazol.

**Objectives:**

This study was performed to compare the ameliorating effects of K-134 and cilostazol on brain damage in an experimental photothrombotic cerebral infarction model.

**Methods and Results:**

We investigated the effects of oral preadministration of PDE3 inhibitors in a rat stroke model established by photothrombotic middle cerebral artery (MCA) occlusion. K-134 significantly prolonged MCA occlusion time at doses >10 mg/kg, and reduced cerebral infarct size at 30 mg/kg in the stroke model (*n* = 12, 87.5±5.6 vs. 126.8±7.5 mm^3^, *P*<0.01), indicating its potent antithrombotic effect. On the other hand, the effects of cilostazol on MCA occlusion time and cerebral infarct size are relatively weak even at the high dosage of 300 mg/kg. Furthermore, K-134 blocked rat platelet aggregation more potently than cilostazol *in vitro*. Also in an arteriovenous shunt thrombosis model, K-134 showed an antithrombotic effect greater than cilostazol.

**Conclusions:**

These findings suggest that K-134, which has strong antithrombotic activity, is a promising drug for prevention of cerebral infarction associated with platelet hyperaggregability.

## Introduction

The underlying pathophysiology in most cases of ischemic stroke involves thrombotic or thromboembolic arterial occlusion [1]. Platelets play a role in the development of atherosclerotic lesions and in thrombus formation following plaque rupture or erosion [2], and antiplatelet therapy is recommended to reduce the risk of recurrent stroke and other cardiovascular events in patients with noncardioembolic ischemic stroke or transient ischemic attack (TIA) [3]. The results of a recent randomized trial, the second Cilostazol Stroke Prevention Study (CSPS 2), indicated that a phosphodiesterase (PDE)3 inhibitor cilostazol is superior to acetylsalicylic acid (ASA) for secondary stroke prevention [4]. K-134 was identified as a more selective PDE3 inhibitor than cilostazol [5], and shown to more potently inhibit human platelet aggregation and thrombus formation [6]. Thus, K-134 is also expected to prevent brain infarction, and it is of interest to compare the effects of K-134 with those of cilostazol.

In non-clinical studies, cilostazol has been shown to attenuate brain injury induced by middle cerebral artery (MCA) occlusion [7], [8]. The pathophysiological mechanism of photothrombotic stroke model is more similar to that of human cerebral infarction than mechanical occlusion models, because photothrombotic MCA occlusion is induced by platelet-rich thrombi following photochemical reaction of rose bengal that causes endothelial damage [9], [10]. In fact, Umemura et al. reported that preadministration of clopidogrel, which is a potent inhibitor of ADP-induced platelet activation, prolonged the time to produce thrombotic occlusion of the MCA and induced a significant reduction in the size of ischemic cerebral damage in this model [10]. To date, little is known about whether K-134 shows more potent antithrombotic activity in *in vivo* thrombotic models and beneficial effects on the photothrombotic stroke model compared to cilostazol. Hence, the aim of the present study was to further evaluate and compare the effects of oral preadministration of K-134 and cilostazol on MCA occlusion and infarct volume in the photothrombotic stroke model.

## Materials and Methods

### Ethics Statement

All study protocols were reviewed and approved by the Committee on Ethics of Animal Experiments of Kowa Company, Ltd.. All surgery was performed under anesthesia with sodium pentobarbital, ether or urethane, and all efforts were made to minimize suffering.

### Drugs and animals

PDE3 inhibitors K-134 (molecular weight (MW) = 399.48 g/mol), cilostazol (MW = 369.46 g/mol), OPC-13015 (MW = 367.44 g/mol) and OPC-13213 (MW = 385.46 g/mol) were obtained from Kowa Company Ltd. (Tokyo, Japan), and dissolved in dimethylformamide for *in vitro* pharmacological experiments or in methanol for preparing standard solution in pharmacokinetic studies, or suspended in 1% (w/w) hydroxypropylmethyl cellulose aqueous solution (HPMC; Shin-Etsu Chemical Co., Ltd., Tokyo, Japan) for *in vivo* experiments. Male Sprague–Dawley (SD) rats and male ICR mice, 5–8 weeks old, were purchased from Japan SLC Inc. (Shizuoka, Japan) and CLEA Japan, Inc. (Tokyo, Japan).

### Photothrombotic MCA occlusion

Brain infarction was produced by photothrombotic MCA occlusion essentially as reported previously [10]. K-134 and cilostazol were administered to SD rats 2 h and 3 h before irradiation for MCA occlusion, respectively (*n* = 12), because K-134 reached plasma peak levels at 1–2 h [11], and cilostazol and its active metabolites (OPC-13015 and OPC-13213) reached at >3 h (Table S1) after oral administration in conscious male SD rats under fed conditions. Rats were anesthetized by intraperitoneal administration of 50 mg/kg of sodium pentobarbital (Kyoritsu Pharmaceutical Co., Ltd., Nara, Japan). Then, a polyethylene catheter for administration of rose bengal was placed in the left femoral vein. Rats were mounted on a brain stereotactic apparatus, and body temperature was maintained at 37°C with a heating pad. The left temporalis muscle was partially cut after incision of the scalp, and craniotomy was performed using a dental drill under an operating microscope. The fiber of a xenon lamp (Model L-4887; Hamamatsu Photonics Co., Ltd., Shizuoka, Japan) was placed on the MCA for irradiation (wavelength: 540 nm, 800000 lux), and the probe of the laser Doppler flow meter (ALF21R; Advance Co., Ltd., Tokyo, Japan) was placed on the MCA distal to the fiber. After 2 min of irradiation, rose bengal (25 mg/kg/mL; Sigma Aldrich Co., MO, USA) was injected for the next 2 min. The MCA was continuously irradiated for 8 min. Blood flow was measured for 18 min from the commencement of irradiation. The MCA occlusion time was determined from blood flow data. When blood flow continued beyond the observation period, the occlusion time was regarded as 16 min. After surgery, the incision site was closed with sutures, and rats were returned to their cages.

### Measurement of infarct volume

Twenty-four hours after MCA occlusion, rats were sacrificed by an overdose of pentobarbital and cutting an abdominal vein. The brain was immediately removed, cooled with cold spray (Pip Co., Ltd., Osaka, Japan), and cut into six slices (2 mm thick) using a slicer (RBSC-02; Neuro Science Inc., Tokyo, Japan) and razor (FA-10; Feather Razor, Co., Ltd., Osaka, Japan). The cutting base point (0 mm) was set at the position of bregma, and brains were cut at −2, −4, 0, +2, +4, +6, +8 mm from the base point. The brain slices were stained with 1% (w/v) 2,3,5-triphenyl-2H-tetrazolium chloride (TTC; Tokyo Chemical Industry Co., Ltd., Tokyo, Japan) in saline solution at 37°C for 20 min. The stained slices were photographed with a digital camera (CAMEDIA E-10; Olympus Co., Ltd., Tokyo, Japan). Whole brain areas and infarcted areas were quantified by image analysis (Win ROOF; Mitani Co., Ltd., Tokyo, Japan), and infarct volume were calculated with the trapezoidal rule.

### Analysis of antiplatelet effects in vitro

Blood was collected via the inferior vena cava under ether anesthesia from SD rats and ICR mice which were fasted overnight, and anticoagulated with a 1/10 volume of sodium citrate (3.0% (w/v) for rats, 3.8% for mice). Platelet-rich plasma (PRP) was prepared by centrifugation (900 rpm, 15 min, at room temperature) of blood (05PR-22 centrifuge with a 03 rotor; Hitachi, Ltd., Tokyo, Japan), and the platelet count was adjusted to 5×10^5^/µL with platelet-poor plasma prepared by centrifugation (3000 rpm, 10 min, at room temperature). PRP was incubated with antiplatelet agents at 37°C for 3 min and stimulated with collagen (final concentration, 15 µg/mL; MC Medical Inc., Tokyo, Japan) or adenosine diphosphate (ADP) (final concentration, 7.5 µM; MC Medical Inc.) (*n* = 5). Platelet aggregation was quantified by measuring the area under the curve (AUC) during the 5-min period after addition of trigger using a light transmittance aggregometer (PAM-8C; Mebanix Co., Ltd., Tokyo, Japan).

### Arteriovenous shunt thrombosis model

Immediately after a single oral administration of the test compound, SD rats were anesthetized by subcutaneous injection of 1.2 g/kg of urethane (*n* = 12). Ninety minutes after oral administration of a single dose of K-134 or cilostazol, one end of a polyethylene catheter (size 3, 24 cm; Hibiki, Tokyo, Japan) filled with physiological saline was inserted into the left jugular vein, with the other end of the catheter inserted into the right common carotid artery to create a loop. The end of the catheter was removed from the jugular vein 4 h after creation of the loop. A thrombus was then regarded to have been formed when blood no longer ran from the catheter. In a separate experiment, blood samples were taken from the inferior vena cava in a rat arteriovenous shunt model under the same conditions as in the above method to create an arteriovenous shunt and heparinized at 0.5, 1.5, 3.5, and 5.5 h after oral administration of a single dose of K-134 or cilostazol (*n* = 6). The serum concentrations of K-134 and cilostazol were determined by high-performance liquid chromatography (HPLC).

### Statistical analyses

Values are expressed as means ± standard error of the mean (SEM). All statistical analyses were performed using SAS 9.1.3 (SAS Institute Japan Ltd., Tokyo, Japan). In all analyses, *P*<0.05 was taken to indicate statistical significance.

## Results

### K-134 reduced cerebral infarction volume through its antithrombotic effects in a photothrombotic stroke model more potently than cilostazol

**Figure 1:**
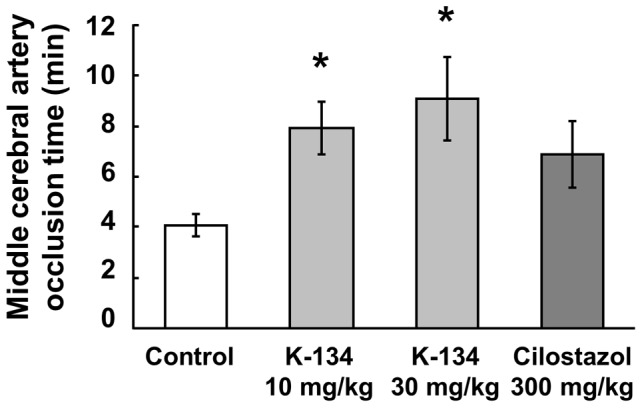
Inhibitory effects of K-134 on middle cerebral artery occlusion time in a photothrombotic stroke model. Preadministration of K-134 but not cilostazol significantly prolonged MCA occlusion time in a photothrombotic cerebral infarction model in comparison with vehicle-treated controls. Values are means ± SEM (*P<0.05, two-tailed Dunnett's test, *n* = 12).

**Figure 2:**
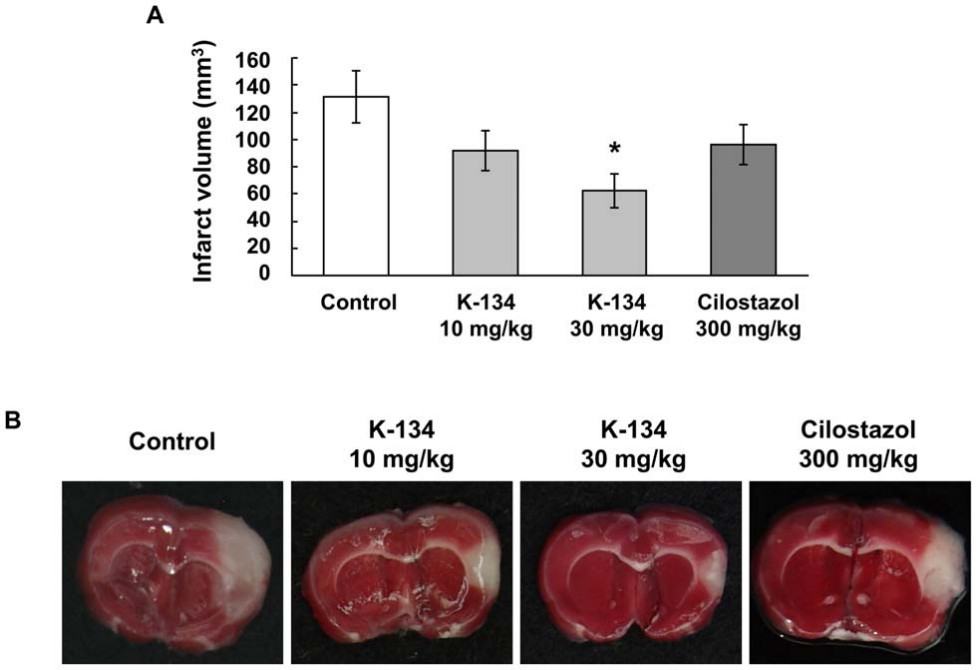
Inhibitory effects of K-134 on infarction volume in a photothrombotic cerebral infarction model. Preadministration of K-134 but not cilostazol significantly and reduced cerebral infarction volume in a photothrombotic cerebral infarction model in comparison with vehicle-treated controls (**A**). Values are means ± SEM (*P<0.05, two-tailed Dunnett's test, *n* = 12). (**B**) Representative photographs (1.35 cm×1.65 cm) show TTC-stained brain sections sliced at the location of bregma. The white-colored area represents the infarct region. Data shown are representative of two experiments with similar results.

The inhibitory effects of PDE3 inhibitors on thrombus formation in photothrombotic stroke model were investigated by evaluating MCA occlusion time. Groups treated with K-134 at 10 and 30 mg/kg showed significantly prolonged MCA occlusion time compared to the control group (7.9±1.1 and 9.1±1.6 min vs. 4.1±0.4 min, respectively) (Fig. 1). In contrast, the effect of high-dose cilostazol (300 mg/kg) on MCA occlusion time was relatively weak and was not statistically significant (6.9±1.3 min) (Fig. 1). Moreover, infarct volume of the 30 mg/kg K-134-treated group was significantly smaller (62.1±12.9 mm^3^) than that of control group (131.4±18.9 mm^3^) (Fig. 2A,B). On the other hand, infarct volume of the groups treated with 10 mg/kg K-134 and 300 mg/kg cilostazol (92.0±14.7 mm^3^ and 95.8±14.8 mm^3^, respectively) were not significantly different from that of the control group (Fig. 2A,B). No bleeding in the cerebrum in rats treated with K-134 and cilostazol was observed macroscopically.

### K-134 inhibited platelet aggregation more potently than cilostazol

**Figure 3:**
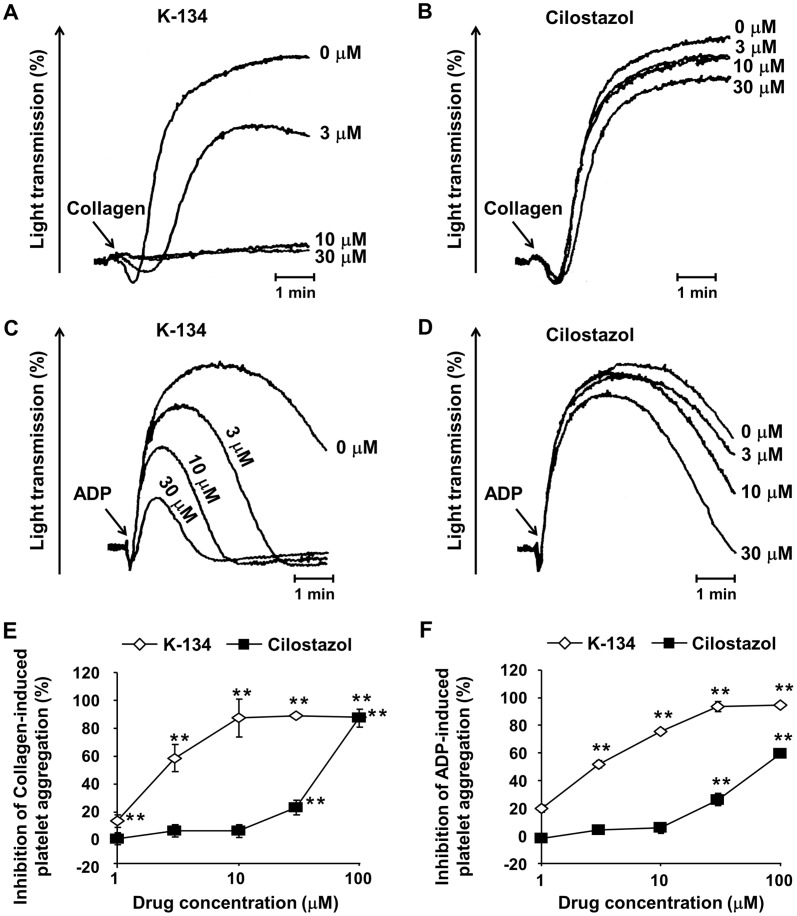
Inhibitory effects of PDE3 inhibitors on rat platelet aggregation *in vitro.* (**A–D**) Representative aggregometer traces showing the effects of PDE3 inhibitors on rat platelet aggregation. Citrated PRP derived from rats was preincubated with K-134 (**A,C**) or cilostazol (**B,D**), and stimulated with collagen (**A,B**) or ADP (**C,D**). Inhibitory effects of agents on collagen (**E**) and ADP (**F**) were estimated by measuring the AUC during the 5 min after stimulation. Values are means ± SEM (*P<0.05, **P<0.01, two-tailed Dunnett's test, *n* = 5).

K-134 and cilostazol inhibited rat platelet aggregation induced by collagen and ADP in a dose-dependent manner *in vitro*. The half-maximal (50%) inhibitory concentration (IC50) values of K-134 were 2.5 µM and 3.2 µM, respectively, and those of cilostazol were 42 µM and 83 µM, respectively (Fig. 3). Furthermore, we previously reported that a single administration of K-134 at a dose of 30 mg/kg resulted in about 55% and 79% inhibition of ADP- and collagen-induced platelet aggregation *ex vivo* in rats, respectively [11]. On the other hand, the inhibitory percentages of cilostazol at a dose of 300 mg/kg were 27% and 50%, respectively (Fig. S1). In *in vitro* experiments, K-134 also inhibited mouse platelet aggregation induced by collagen and ADP in a dose-dependent manner, and the IC50 values were 5.5 µM and 6.7 µM, respectively (Fig. S2). Next, we assessed the overall bleeding risk of K-134 in general in mice. Single oral administration of K-134 did not prolong bleeding time at a dose of 30 mg/kg compared to control (106±5 vs. 110±5 s, not significant) (Fig. S3). Moreover, we detected a sufficiently high enough plasma concentration of K-134 (13.6±2.3 µM) to inhibit platelet aggregation at 10 min after single administration in mice at a dose of 30 mg/kg, which is the same time point as the above test of bleeding time (Table S2).

### K-134 showed greater antithrombotic activity than cilostazol in an arteriovenous shunt thrombosis model

**Figure 4:**
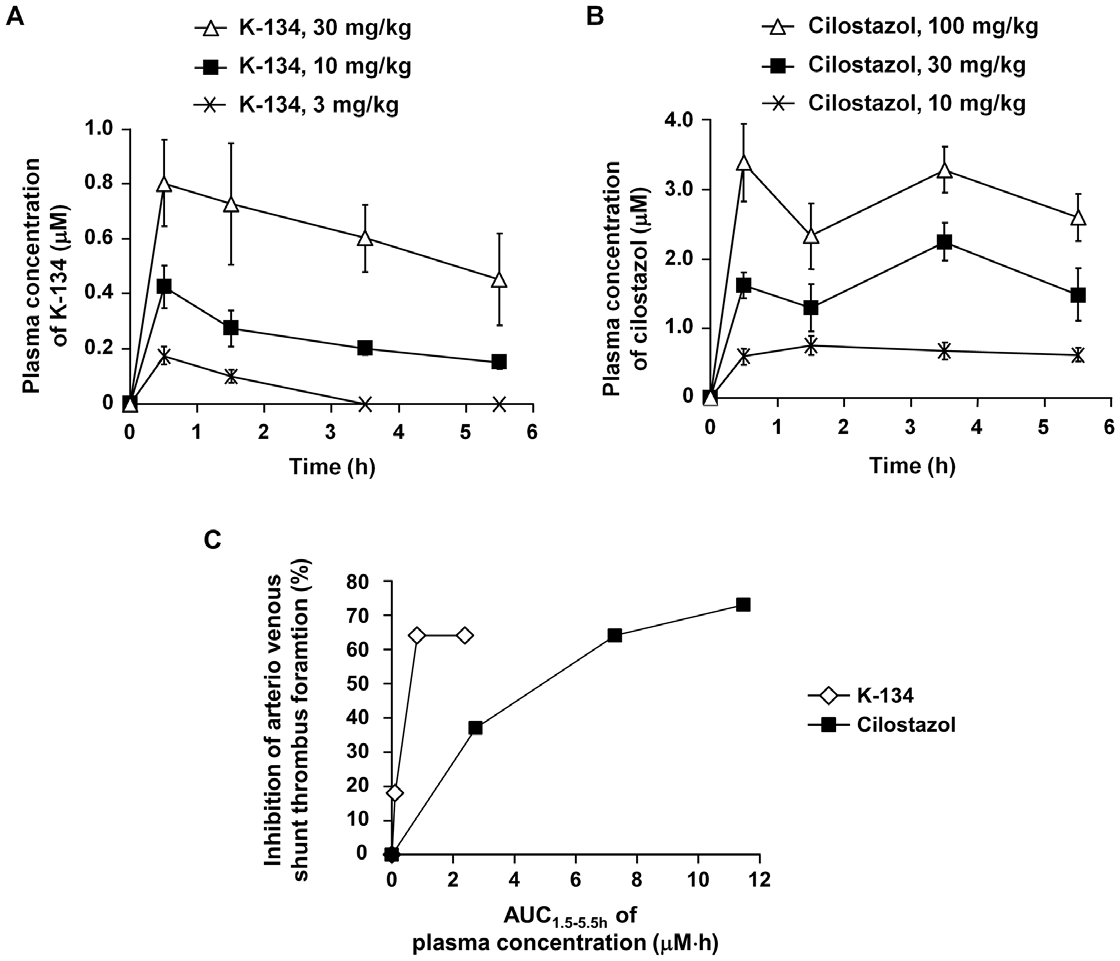
Plasma concentration of K-134 and cilostazol in a rat arteriovenous shunt model. Blood samples were taken at 0.5, 1.5, 3.5, and 5.5 h after oral drug administration in a rat arteriovenous shunt model under the same conditions as described in Table 1, and plasma concentrations of K-134 (**A**) and cilostazol (**B**) were determined by HPLC. Values represent means ± SEM of the plasma concentration (µM) (*n* = 6). (C) The dose-response curve between the area under the plasma drug concentration-time curve from time 1.5 h to 5.5 h (AUC_1.5–5.5 h_) and the inhibition rate of arteriovenous shunt thrombus formation. AUC_1.5–5.5 h_ was estimated from data of Fig. 4A and 4B.

Next, the effects of PDE3 inhibitors on thrombus formation were also investigated in an arteriovenous shunt model in rats. K-134 significantly reduced the incidence of occlusive shunt thrombi at doses above 10 mg/kg (half-maximal effective dose: ED50 = 11 mg/kg). On the other hand, cilostazol significantly reduced the incidence of occlusive shunt thrombi at doses above 30 mg/kg (ED50 = 18 mg/kg) (Table 1). The plasma concentration of K-134 was 0.43±0.08 µM (Cmax) at a dose of 10 mg/kg, while that of cilostazol was 2.08±0.28 µM at a dose of 30 mg/kg (Fig. 4). We calculated the area under the plasma drug concentration-time curve from time 1.5 h to 5.5 h (AUC_1.5–5.5 h_) using data of Fig. 4A and 4B for estimating the relative efficiency of drugs when arteriovenous shunt loops were exposed to flowing blood on the fact that K-134 and cilostazol are reversible PDE3 inhibitors. The dose-response curve between the AUC_1.5–5.5 h_ and the inhibition rate of arteriovenous shunt thrombus formation indicates that K-134 inhibited arteriovenous shunt thrombus formation at a lower plasma concentrations compared with cilostazol (Fig. 4C).

**Table 1:** Inhibitory effects of PDE3 inhibitors on arteriovenous shunt thrombus formation.

	Control	K-134 (mg/kg)			Cilostazol (mg/kg)		
		3	10	30	10	30	100
Occluded/Total	11/12	9/12	4/12*	4/12*	7/12	4/12*	3/12**
Occlusion (%)	92	75	33	33	58	33	25
Inhibition (%)	N/A	18	64	64	37	64	73
ED_50_ (mg/kg)	N/A	11			18		

K-134 and cilostazol were administered to rats in a single dose 90 min before creation of the arteriovenous shunt. K-134 at concentrations above 10 mg/kg and cilostazol at concentrations above 30 mg/kg decreased the incidence of occlusive arteriovenous shunt thrombi (%) (*P<0.05, *P<0.01, two-tailed Fisher's exact test with Bonferroni's procedure, *n* = 12).

## Discussion

The present study provided novel data indicating that K-134 blocks brain injury more potently than cilostazol in a photothrombotic ischemic stroke model (Fig. 2) and other thrombosis models (Table 1, Fig. 4). The IC50 values of K-134 (also known as OPC-33509) toward PDE2, PDE3A, PDE3B, PDE4, and PDE5 are >300, 0.10, 0.28, >300, and 12.1 µM, respectively, while those of cilostazol are 45.2, 0.20, 0.38, 88.0, and 4.4 µM, respectively [5]. As platelets mainly express PDE3A [12], the above beneficial effects of K-134 compared with cilostazol can be partly attributed to the more selective inhibitory effect of K-134 on PDE3A than cilostazol. In fact, we showed that K-134 inhibits platelet aggregation in rats *in vitro* and *ex vivo* with greater potency than cilostazol.

K-134 exerts a similar dose-dependent vasodilatory effects on rat femoral arteries contracted by KCl *in vitro* [11] compared with cilostazol (Fig. S4), and both drugs increases both pre- and post-treadmill exercise hindlimb blood flow after 1 week of treatment to a similar extent in a rat experimental peripheral artery disease model [13]. In contrast, anti-platelet activities of K-134 are more potent than cilostazol. K-134 is currently being developed for treating intermittent claudication associated with peripheral arterial diseases [14] and the beneficial effects of PDE3 inhibitors on peripheral arterial disease are attributable to not only antiplatelet activity but also vasodilatory activity or some chronic unknown effects [11], [13], as no studies have shown a benefit of other antiplatelet drugs such as acetylsalicylic acid and clopidogrel [15]. Meanwhile, the potent anti-platelet activity of K-134 also make it a potential alternative agent for preventing secondary cerebral infarction because antiplatelet therapy for secondary stroke prevention has been proved to be beneficial in clinical trials [3]. Moreover, a double-blind, randomized trial of cilostazol and aspirin demonstrated that cilostazol is non-inferior, and might be superior to aspirin for prevention of stroke after an ischemic stroke [4]. For these reasons, we used cilostazol as a comparative drug to evaluate the efficacy of K-134 on the photothrombotic stroke model in this study.

In the stroke model, 30 mg/kg of K-134 inhibited photothrombotic MCA occlusion and reduced cerebral infarct size more potently than 300 mg/kg of cilostazol (Fig. 1,2). On the other hand, the lower dose (10 mg/kg) of K-134 significantly prolonged MCA occlusion time and tended to decrease infarct volume, but this decrease was not statistically significant. This difference of effects might be due to a short half-life (T_1/2_ = about 2 h) of K-134 when administered to rats at a dose of 10 mg/kg [11], because MCA occlusion time and infarct volume were evaluated 2 and 24 hours after administration of K-134, respectively. Thus, 10 mg/kg of K-134 may not have been able to inhibit platelet thrombus formation induced by endothelial injury during the later hours of treatment.

The photothrombotic MCA occlusion model has numerous advantages for the study of antiplatelet inhibitors *in vivo* because this model permits observation of not only time to occlusion by thrombus formation but also effects on cerebral infarction at a certain period of time after endothelial injury by less-invasive approach without injuring dura mater, thereby enables taking account of effects of rate of drug metabolism and long-term pharmacological effects of drugs. We previously reported that K-134 blocks stable platelet accumulation but not initial platelet adhesion onto Von Willebrand factor (vWF)-coated surface under high shear conditions *in vitro* [6], but we could not determine whether K-134 can inhibit initial platelet adhesion to a damaged blood vessel in this *in vivo* model. Hence, intravital videomicroscopy analysis [16] is needed to reveal the detailed mechanisms of antiplatelet action of K-134 *in vivo*.

The photothrombotic stroke model is a suitable model for evaluating the effects of antiplatelet inhibitors because an arterial platelet aggregation is induced through endothelial damage by a photochemical reaction [17]. However, we could not test the possibility that other effects of K-134 such as cerebral blood flow increase via its vasodilatory activity [11] contribute to decrease of infarct volume in this model. Besides, true stroke has many causes other than platelet activation. Hence, care should be taken in interpreting the results obtained in the photothrombotic stroke model in the present study in terms of stroke prevention in humans, and additional studies using other stroke models, such as Stroke-Prone Spontaneously Hypertensive Rats (SHRSP) [18], are necessary to obtain further insight into the therapeutic benefits of PDE3 inhibitors. Moreover, comparative studies of the effects of K-134 and other antiplatelet agents such as aspirin and P2Y12 inhibitors on stroke models are required to further extend our findings.

In the photothrombotic cerebral infarction model, photoactivation of rose bengal by illumination with green light results in reactive oxygen intermediates (predominantly singlet oxygen and superoxide). In our electron spin resonance (ESR) experiments, K-134, cilostazol, and OPC-13015 (one of cilostazol metabolites) did not show scavenging activities against both singlet oxygen and superoxide anion radical (data not shown). Therefore, we concluded that the observed inhibitory effects of K-134 on photothrombosis in this model are relevant to its antiplatelet activity but not radical scavenging activity.

In the case of oral administration of cilostazol in *in vivo* experiments, antiplatelet activities of its active metabolites, OPC-13015 and OPC-13213, could also contribute to inhibition of platelet thrombus formation. OPC-13015 has 3 times more potent antiplatelet activity than cilostazol, whereas OPC-13213 has 3 times less potent activity than cilostazol [19]. Cmax of cilostazol, OPC-13015 and OPC-13213 were 2.4, 1.4 and 9.1 µM, respectively, and AUC_0–24 h_ were 30.1, 18.3, and 127.3 µM·h, respectively, after a single oral administration of cilostazol at a dose of 300 mg/kg in non-fasting male SD rats (Table S1). On the other hand, the results of the healthy male single dose study showed that in human after administration of 100 mg of cilostazol, Cmax of cilostazol, OPC-13015 and OPC-13213 were about 625, 122 and 64 µg/L (equal to 1.69, 0.33 and 0.17 µM), respectively, and AUC_0–72 h_ were about 8087, 2423 and 617 µg/L·h (equal to 21.89, 6.59 and 1.60 µM·h), respectively [20]. On the basis of these results, we concluded that the dosage (300 mg/kg) of cilostazol used in the rat photothrombotic stroke model in our study could give sufficient plasma concentrations of cilostazol and its metabolites compared with their therapeutic plasma concentrations in human. In contrast to cilostazol, AUC_0–24 h_ of K-134 after a single oral administration to non-fasting male SD rats at doses of 10 mg/kg and 30 mg/kg were low (2.38 and 14.09 µM·h, respectively) because of its short elimination half-life (T_1/2_) compared with that of cilostazol [11]. Therefore, development of an extended release oral dosage form of K-134 may lead to greater long-term pharmacological effects *in vivo*. In the arteriovenous shunt thrombosis model, K-134 showed antithrombotic activity at a dose of 10 mg/kg which gives much lower plasma concentration compared with that of cilostazol at an effective dose of 30 mg/kg (Table 1 and Fig. 4). Moreover, we demonstrated that K-134 at a dose of 30 mg/kg showed more beneficial effects compared with cilostazol at a higher dose of 300 mg/kg in the rat cerebral infarction model (Fig. 1, 2). Taken together, the potent antiplatelet activity of K-134 exceeds total antiplatelet activities of cilostazol and its metabolites *in vivo*.

In contrast to the evidence from clinical trials and guidelines supporting antiplatelet therapies for secondary stroke prevention [3], the benefit of antiplatelet agents for primary stroke prevention has not been satisfactorily proven in patients with diabetes [21]. Moreover, anticoagulation therapy with warfarin is substantially more efficacious than antiplatelet therapy with aspirin for primary stroke prevention in patients who have nonvalvular atrial fibrillation [22]. For these reasons, we assume that K-134 is expected to be developed for secondary stroke prevention than for primary prevention.

In summary, K-134 significantly prevented brain damage by inhibiting thrombus formation in the rat cerebral infarction model. This effect was attributable to potent antiplatelet activity of K-134. Recurrence of ischemic stroke is involved in platelet activation. Therefore, K-134 is a promising drug for secondary prevention of ischemic stroke due to its potent inhibitory activity on platelet thrombus formation.

## Supporting Information

Figure S1 **Antiplatelet effects of cilostazol** ***ex vivo*** **in rats.** Antiplatelet effects of cilostazol were investigated ex vivo. Three hours after administration of cilostazol, blood was collected for preparing platelet-rich plasma. Platelet aggregation was induced by ADP (A) or collagen (B), and inhibitory effects were estimated by measuring the AUC during the 5 or 7 min after addition of ADP or collagen, respectively. Values are means ± SEM (*P<0.05, ***P<0.001 vs. vehicle control, two-tailed Dunnett's test, n = 8).
(TIF)

Figure S2 **Inhibitory effects of PDE3 inhibitors on mouse platelet aggregation** ***in vitro.*** Citrated PRP derived from mice was preincubated with K-134 or cilostazol, and stimulated with collagen (A) or ADP (B). Inhibitory effects of agents on collagen (A) and ADP (B) were estimated by measuring the AUC during the 5 min after stimulation. Values are means ± SEM (*P<0.05, **P<0.01, two-tailed Dunnett's test, n = 5).
(TIF)

Figure S3 **Bleeding time in mice.** K-134 and warfarin were administered to mice at 10 min and 24 h before testing of tail bleeding time, respectively. Values are means ± SEM (n = 27–28). **P<0.01 vs. vehicle control by two-tailed t-test. N.S. = not significantly different by two-tailed Dunnett's test.
(TIF)

Figure S4 **Vasodilatory effects of cilostazol.** Vasodilatory effects of cilostazol were evaluated as previously described [11]. Cilostazol was added cumulatively to rat femoral arteries pre-contracted by KCl. Each point represents the mean ± SEM of percent vasodilation (n = 3).
(TIF)

Table S1 **Pharmacokinetic parameters of cilostazol and its active metabolites (OPC-13015 and OPC-13213) in rats.**
(DOC)

Table S2 **Plasma concentration of K-134 in mice.**
(DOC)

Methods S1 **Description of pharmacokinetic studies,** ***ex vivo*** **antiplatelet effects study, vasodilation study and bleeding time study.**
(DOC)
