# Supplementary material for: K-134, a Phosphodiesterase 3 Inhibitor, Prevents Brain Damage by Inhibiting Thrombus Formation in a Rat Cerebral Infarction Model
Source: PLoS One. 2012 Oct 23;7(10):e46432. doi: 10.1371/journal.pone.0046432 (PMC3479105; doi:10.1371/journal.pone.0046432)
Supplement: Table S1 — Pharmacokinetic parameters of cilostazol and its active metabolites (OPC-13015 and OPC-13213) in rats. (DOC) [file pone.0046432.s005.doc]

**Table S1.**

**Pharmacokinetic parameters** of cilostazol and its active metabolites (OPC-13015 and OPC-13213) in rats

|  | **Cmax (μM)** | **AUC_0-24h_ (μM·h)** | **Tmax (h)** | **T_1/2_ (h)** |
| --- | --- | --- | --- | --- |
| **Cilostazol** | 2.4 ± 0.2 | 30.1 ± 2.0 | 5.8 | 5.4 |
| **OPC-13015** | 1.4 ± 0.1 | 18.3 ± 1.4 | 3.2 | 6.8 |
| **OPC-13213** | 9.1 ± 1.3 | 127.3 ± 18.3 | 7.0 | 6.1 |

Pharmacokinetic variables (Cmax, AUC, Tmax and T_1/2_) were calculated from the plasma drug concentration-time curve. Cilostazol was orally administered to rats under non-fasting conditions at a single dose of 300 mg/kg to determine the concentration-time profile in serum (n = 3). Values are means (± SEM). AUC_0–24h_ indicates the AUC from time zero to 24 h.
