# Supplementary material for: K-134, a Phosphodiesterase 3 Inhibitor, Prevents Brain Damage by Inhibiting Thrombus Formation in a Rat Cerebral Infarction Model
Source: PLoS One. 2012 Oct 23;7(10):e46432. doi: 10.1371/journal.pone.0046432 (PMC3479105; doi:10.1371/journal.pone.0046432)
Supplement: Table S2 — Plasma concentration of K-134 in mice. (DOC) [file pone.0046432.s006.doc]

**Table** **S2**.

Plasma concentration of K-134 in mice

| **Dosage of K-134** | **Plasma concentration of K-134** |
| --- | --- |
| 1 mg/kg | 0.4 ± 0.0 μM |
| 3 mg/kg | 1.1 ± 0.2 μM |
| 10 mg/kg | 4.3 ± 0.6 μM |
| 30 mg/kg | 13.6 ± 2.3 μM |

Plasma concentration of K-134 was measured at 10 min after a single oral administration of K-134 at doses of 1, 3, 10, and 30 mg/kg to male ICR mice. Values are means ± SEM (n = 6).
