# Supplementary material for: K-134, a Phosphodiesterase 3 Inhibitor, Prevents Brain Damage by Inhibiting Thrombus Formation in a Rat Cerebral Infarction Model
Source: PLoS One. 2012 Oct 23;7(10):e46432. doi: 10.1371/journal.pone.0046432 (PMC3479105; doi:10.1371/journal.pone.0046432)
Supplement: Methods S1 — Description of pharmacokinetic studies, ex vivo antiplatelet effects study, vasodilation study and bleeding time study. (DOC) [file pone.0046432.s007.doc]

**Supplementary Methods**

***Pharmacokinetic studies***

We measured plasma concentration of cilostazol and its active metabolites, OPC-13015 and OPC-13213 at 0.5, 1, 2, 3, 4, 6, 8, and 24 h after a single oral administration of cilostazol to non-fasting male SD rats (8 weeks old) at a dose of 300 mg/kg using high-performance liquid chromatography (HPLC), and calculated their pharmacokinetic parameters (Cmax, Tmax, T_1/2_, and AUC) in noncompartmental analysis using WinNonlin (Version 4.0.1, Pharsight Corporation, CA, USA). We also measured plasma concentration of K-134 at 10 min after a single oral administration of K-134 to male ICR mice (Japan SLC Inc., 5 weeks old).

***Analysis of the antiplatelet effects ex vivo in rats using an aggregometer***

Cilostazol was orally administered to rats under non-fasting conditions in a single dose. Then, 3 h after administration, blood was collected from the inferior vena cava and anticoagulated with a 10% volume of 3.8% (w/v) sodium citrate. Platelet-rich plasma (PRP) was prepared by centrifugation (450 × g, 8 min, 25°C) of blood. PRP was stimulated with adenosine diphosphate (ADP) (final concentration = 4 μM; MC Medical Inc., Tokyo, Japan) or collagen (final concentration = 5 μg/mL; MC Medical Inc.). Platelet aggregation was quantified by measuring the area under the curve (AUC) during the 5 or 7 min after addition of ADP or collagen, respectively, using a light transmittance aggregometer (Hema Tracer 313M; MC Medical Inc.).

***Vasodilation study***

Femoral artery rings (3 mm in width) prepared from rats were placed in a magnus tube filled with Krebs–Henseleit solution at 37°C and bubbled continuously with a gas mixture of 95% O_2_ and 5% CO_2_ as described previously [11]. The isometric tension of the rings was measured using a force displacement transducer (T7-15-240; A&G Co., Ltd., Fukuoka, Japan) and recorded with a thermal array recorder (WR-300; Graphtec Corp., Tokyo, Japan). The rings were equilibrated at a resting tension of 0.75 g. After contraction induced by 60 mM KCl, cilostazol was added cumulatively to the magnus tube. At the end of each experiment, 100 μM papaverine was added to determine the maximum relaxation.

***Analysis of the effects of K-134 on bleeding time***

K-134 and warfarin (Sigma Aldrich Co., MO, USA) were suspended and dissolved in 1% HPMC, respectively, and orally administered to ICR mice at 10 min or 24 h before testing of bleeding, respectively. Each mouse was placed in a restrainer, which exposes the tail. A 27G disposable needle (Terumo Co., Tokyo, Japan) was inserted about 6 mm into a tail vein 2 cm from the tip of the tail. Immediately after the operation, sheets of filter paper were placed under the injury site every 15 sec to absorb the blood, and the time point at which no blood was observed on the filter paper was regarded as the bleeding time.
